# Effects of laughter yoga on health-related quality of life in cancer patients undergoing chemotherapy: a randomized clinical trial

**DOI:** 10.1186/s12906-023-04028-2

**Published:** 2023-06-12

**Authors:** Mohammad Namazinia, Seyyed Reza Mazlum, Samira Mohajer, Violeta Lopez

**Affiliations:** 1 Department of Nursing, School of Nursing and Midwifery, Torbat Heydariyeh University of Medical Sciences, Torbat Heydariyeh, Iran; 2 Department of Medical Surgical Nursing, School of Nursing and Midwifery, Mashhad University of Medical Sciences, Mashhad, Iran; 3 Nursing and Midwifery Care Research Center, Mashhad University of Medical Sciences, Mashhad, Iran; 4 Department of Nursing Science, Faculty of Medicine, University of Malaya, Kuala Lumpur, Malaysia; 5 School of Nursing, Midwifery and Social Sciences, Central Queensland University, Queensland, Australia

**Keywords:** Laughter yoga, Health-Related Quality of Life, Cancer Patients, Chemotherapy

## Abstract

**Background:**

Chemotherapy is associated with a wide range of physical and psychological side effects, so complementary and alternative therapies may be practiced as an independent treatment or combined with the standard ones to improve health-related quality of life of cancer patients. Laughter yoga has predominantly been used as a complementary therapy to enhance health and wellbeing of ordinary people and patients with chronic diseases. However, to date, few studies have evaluated the effects of this modern exercise on cancer patients undergoing chemotherapy in clinical settings, to the best of the authors’ knowledge. the present study aimed to investigate the effects of Laughter Yoga on the health-related quality of life of cancer patients undergoing chemotherapy.

**Methods:**

This study was a two-group randomized clinical trial on 69 cancer patients undergoing chemotherapy at Reza Radiotherapy and Oncology Center, Iran in 2018. Patients were randomly divided into intervention and control groups. The intervention group received laughter yoga for four sessions at one-week intervals. Each session consists of one part and lasts for 20–30 min. Patients’ health-related quality of life was assessed before and after the laughter yoga sessions using Quality of Life Questionnaire European Organization for Research and Treatment of Cancer (EORTC QLQ-C30) version 3.0. SPSS Statistics (v.20 software was used to conduct Chi-square, independent t-test, Mann-Whitney, Wilcoxon and paired t-tests analyses of the data.

**Results:**

The number of participants in intervention and control groups were 34 and 35, there was no significant difference of demographic and disease related characteristics and pre-intervention HRQOL between two groups. In the intervention group, there is significant difference between pre- and post-intervention scores (Mean ± Standard Deviation) of emotional functioning (12.99 ± 10.49), physical functioning (0.78 ± 6.08), role functioning (3.43 ± 7.97), fatigue (-8.82 ± 22.01), pain (-8.33 ± 11.78), sleep disturbance (-15.68 ± 18.77), and global health and quality of life (6.37 ± 5.04) (p < 0.05). There was no significant change in the control group. Participants reported no adverse events.

**Conclusions:**

A structured laughter yoga intervention in a hospital setting effectively improved health-related quality of life for cancer patients undergoing chemotherapy. Benefits to many patients could be expected if this would become a part of routine care.

**Trial Registration:**

This study was registered in the Iranian Registry of Clinical Trials (no. IRCT20180429039463N1) on 21/08/2018.

**Supplementary Information:**

The online version contains supplementary material available at 10.1186/s12906-023-04028-2.

## Introduction

Cancer accounts for 9% of all deaths across the world, and the second leading cause of mortality in developing nations following cardiovascular diseases [1]. The age-standardized rates in cancer incidence and mortality were estimated to be 152.7 and 94.0, respectively, per 100,000 populations in Iran in 2020 [2, 3]. Most cancer patients receive chemotherapy as a definitive treatment option to increase their life expectancy and survival rate [4–6]. This type of cancer treatment gives rise to an extensive range of physical and psychological impacts related to patients’ health-related quality of life (HRQOL) [7–9]. Health-related quality of life (HRQOL) refers to the state of wellbeing expressed by the participants during the period of illness or treatment with regard to ability to perform daily activities (including physical, mental, and social functions), experience of physical symptoms and difficulties (such as pain, nausea/vomiting, fatigue, sleep disturbance, loss of appetite, financial difficulty, etc.), and perception towards their overall health and quality of life [10–12]. Being endowed with higher HRQOL can thus result in better acceptance and reduced complications in patients once diagnosed [12]. Boosting HRQOL also diminishes the accompanying medical and healthcare costs [13]. For the purpose of mitigating the discomforts associated with the disease and treatment, and then improve HRQOL in patients, complementary and alternative therapy may be utilized as an independent treatment or one combined with standard therapy [14, 15].

Laughter yoga is a type of complementary therapy which also incorporates some other components including mild type of physical exercises. This type of treatment combines unconditional laughter with yoga breathing practices and yoga stretching poses so that patients laugh different from jokes or humor programs [16]. Some scholars believe that both real and fake laughter can have the same effects on the body [17–21]. Laughter yoga, first introduced by an Indian physician, comprised of a number of exercises together with laughter [16]. It includes four main steps: clapping and body movement, deep breathing, childlike playfulness, and laughter exercises. Laughter also triggers the release of endorphins and decreases stress hormones that make a person feel good [17–21]. It means that laughter yoga releases a rush of stress-busting hormones, like epinephrine and dopamine [22]. A good, hearty laugh from the belly also oxygenates the body and provides an emotional and physical release, removing tension and leaving the body relaxed so that makes a person feel good. This modern exercise is also an easy, cost-effective, and affordable method that can maintain mental well-being in patients. It is also possible to teach laughter yoga in patients so that they can practice this by themselves, which helps increase their self-care management [16, 17, 23]. Many studies in Iran and other countries evaluated the effectiveness of laughter yoga to enhance the health and wellbeing of ordinary people and patients with chronic diseases [16, 24–26]. However, there are few studies on its effect among cancer patients undergoing chemotherapy in a clinical setting [14]. According to Namazi-Nia et al., laughter yoga had enhanced mental well-being scores in cancer patients undergoing chemotherapy [23]. In the study by Farifteh et al., laughter yoga in cancer patients showed reduced stress before chemotherapy and elevated HRQOL [27]. To date, in spite of the beneficial effects of this therapeutic technique, little research has been conducted in this field, particularly for improving HRQOL in cancer patients undergoing chemotherapy.

The purpose of this study was to determine the effect of laughter yoga on HRQOL in cancer patients undergoing chemotherapy. We hypothesized that implementing the laughter yoga program will significantly promote HRQOL in patients undergoing chemotherapy.

## Methods

### O trial design

This study was a single-center, two-group randomized clinical trial comparing the effects of structured laughter yoga program in cancer patients before chemotherapy. The study is reported using the CONSORT (Consolidated Standards of Reporting Trials) checklist.

### O participants

The inclusion criteria were cancer patients with an age range of 18–60, having non-metastatic type of cancers, no auditory-visual problems, undergoing four sessions of chemotherapy per month, absence of stomatitis symptoms, no upper gastrointestinal (UGI) cancer, attending no simultaneous radiotherapy programs, as well as the mental and physical ability to perform laughter yoga. The exclusion criteria were chronic stress during the study approved by the psychologist of the center, disease exacerbation and the need for intensive care services, changes in chemotherapy programs due to thrombocytopenia or any other factors, and modifications in chemotherapy drug regimen. This study was a two-group randomized clinical trial on 69 cancer patients undergoing chemotherapy. It was conducted at the at Reza Radiotherapy and Oncology Center, Iran, in 2018. Patients were randomly divided into intervention and control groups., Mashhad, Iran, between October 2018 and June 2019.

### O intervention

The intervention group received laughter yoga for four sessions with one-week intervals. Each session lasts for 20–30 min and it consists of 15 steps of laughter yoga performed consecutively. And each laugh lasts approximately 30 to 45 s. This intervention was provided by researchers who had completed laughter yoga training course from the laughter yoga instructor and participants were supervised during each session. Laughter yoga sessions were held in three groups of 8, 12, and 14 cancer patients. The intervention was carried out before the chemotherapy according to the protocol. The four sessions of the intervention were performed in a standing position following the 15 steps (Supplementary Material 1).

In the control group, only routine self-care training was conducted by the researchers in the meeting hall in the form of face-to-face education and the use of pamphlets. This program was implemented exactly the same for the intervention group with 10 min one session each week for four weeks. The educational content was developed after reviewing the related literature, up-to-date studies, guidelines of the National Cancer Prevention and Control Program published by the Ministry of Health and Medical Education (MHME), World Health Organization [28], and experts in the field of health psychology for cancer patients. The education program contents included infection prevention, oral hygiene, skin and hair health, nausea and vomiting, improved nutrition and fatigue.

### O outcomes

The primary outcome of this trial was HRQOL, which was assessed using EORTC QLQ-C30 (European Organization for the Research and Treatment of Cancer Quality of Life Questionnaire version 3). Demographic information was also collected using a structured questionnaire.

Demographic information questionnaire had 6 questions about age, frequency of chemotherapy, sex, type of cancer, previous chemotherapy experience, and experience of participating in program. This questionnaire was completed through an interview before the intervention.

EORTC tool contained 30 items with five functioning domains, nine symptoms, and Global HRQOL status. HRQOL means subjective feeling of patients regarding their overall health and quality of life. The five functioning domains included physical functioning, role functioning, emotional functioning, cognitive functioning, and social functioning. The nine symptoms included fatigue, nausea/vomiting, pain, shortness of breath, sleep disturbance, appetite loss, constipation, diarrhea, and financial hardship. Raw score obtained from the rating given by the participants on questions was converted into transformed score ranging from zero to 100 as per the scoring manual. A higher value of HRQOL and functional scores, and lower value of symptoms and single items represents better health and wellbeing [29]. This questionnaire had shown good internal reliability in several studies to determine HRQOL in cancer patients [30, 31]. A study found that the Persian version of EORTC QLQ-C30 was a reliable and valid tool and could be used in epidemiological and clinical research studies on cancer. The Cronbach’s alpha coefficient in most domains of the questionnaire was more than 0.7 and the convergence validity was 41–79% [32]. As used in this study, the internal consistency reliability of EORTC QLQ-C30 was Cronbach’s alpha = 0.81.

The study questionnaires were completed before and after the laughter yoga sessions by the cancer patients, through interviews in a quiet room at the meeting hall next to the chemotherapy Center. First baseline demographic data were taken and initial assessment of HRQOL was done. Final assessment of HRQOL was done after four weeks, when laughter yoga intervention was delivered to the intervention group and self-care training education was given to the control group. Participants in both groups received their routine chemotherapy in between the assessments.

### O sample size and randomization

**Fig. 1:**
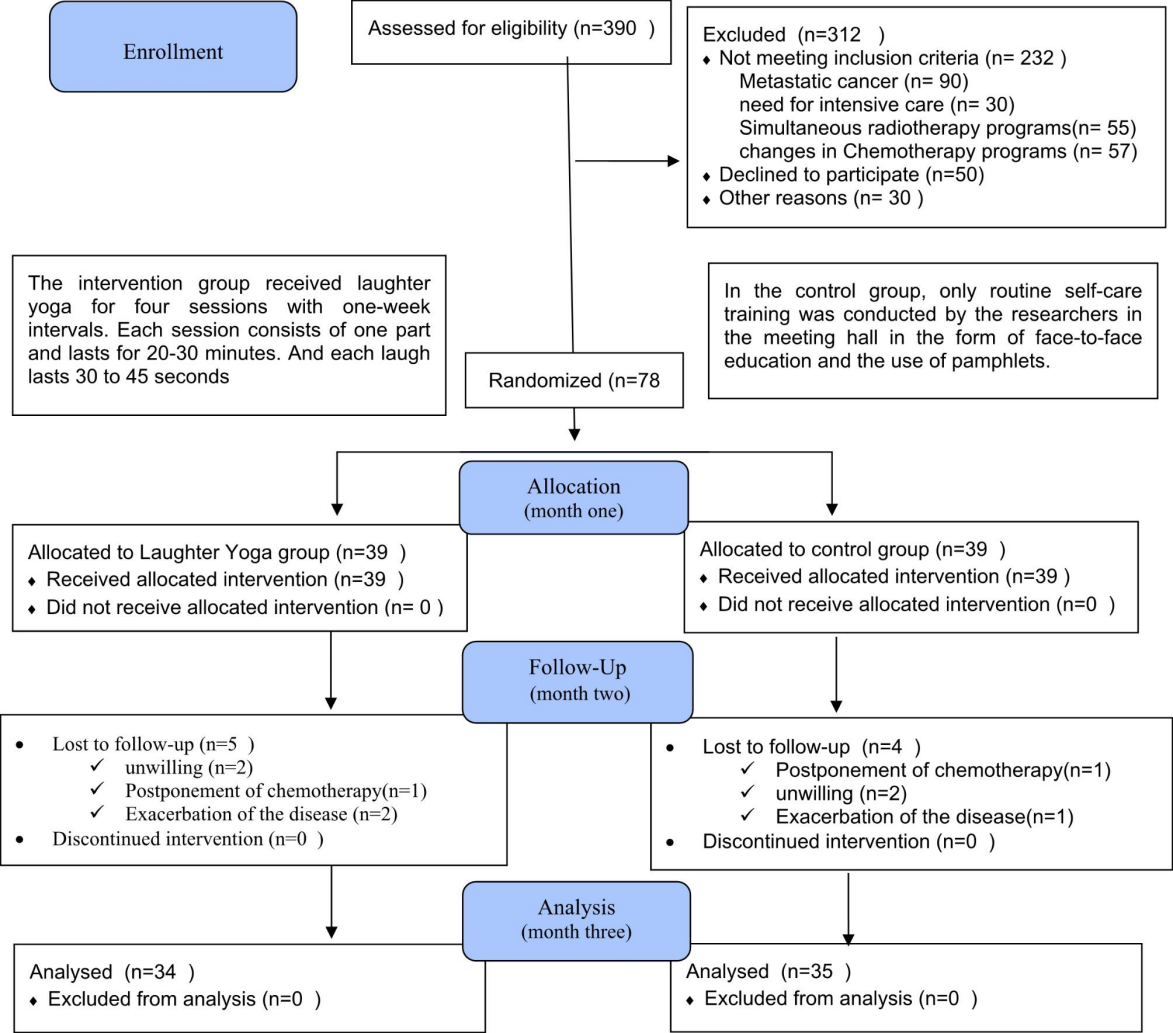
CONSORT Flow Chart of participants

The sample size was determined by 34 patients in each group based on the results of a pilot study on 10 participants in each group using the comparison of two means formula with 95% confidence interval and 80% test power. Assuming the possibility of participants being lost to follow up loss of some cases, and to give more assurance to the completion of study with required sample size, 39 cancer patients in each group were included in the present study. Among them, five cases from the intervention group and four individuals from the controls were lost to follow up. Finally, 69 cancer patients remained in the study (Fig. 1).

The first author of the study, as the research coordinator, referred to the chemotherapy unit and extracted the list of patients undergoing chemotherapy. Then introduced oneself to the cancer patients and selected eligible ones with reference to the mentioned criteria. The cancer patients were selected based on the convenience sampling method and then divided into the intervention and control groups using random sequence generated by the SPSS Statistics (v.20) software, kept in a closed envelope. Upon providing an individual oral explanation about the research objectives and methodology, informed written consent was obtained from the cancer patients in both groups. Although it is difficult to blind the participants in this trial, the outcome assessors and statisticians were blinded to the type of intervention.

### O statistical methods

The data was analyzed using the SPSS Statistics (v.20) software total of 69 cancer patients out of 78 were included in the data analysis as nine cancer patients were lost to follow -up. Questionnaire was checked for completeness just after the participants returned it. The descriptive statistics (viz., frequency distribution, mean, and standard deviation) were used to describe and categorize the data. Inferential statistics including the Chi-square test, independent-samples t-test, and Mann-Whitney U test were used to test the research hypothesis. Wilcoxon signed-rank test and paired-samples t-tests were further employed for intra-group comparisons. The normality of the quantitative variables was correspondingly assessed by the Kolmogorov-Smirnov test. The significance level of 0.05 was set for all the tests in this study.

## Results

390 patients were assessed for eligibility. Once the desired sample size was reached, recruitment efforts ended. The 78 eligible candidates were randomly allocated into intervention (n = 39) and control (n = 39). The final number of participants available for comparison of baseline and flow up data was 69. The number of participants lost to follow up was 5 in the intervention group and 4 in control group. Thus, the number of participants in the final analysis was 34 in intervention group and 35 in control group (Fig. 1).

The majority of patients in the intervention (n = 22, 67.7%) and control (n = 24, 68.6%) groups were females. There was no significant difference between two groups in terms of other demographic and disease data (p > 0.05) (Table 1).

**Table 1:** General baseline characteristics of the participant’s

Variables		Groups		P-value
		Intervention	Control	
Age (mean ± SD)		49.0 ± 9.6	45.2 ± 12.6	P = 0.378***
Frequency of chemotherapy(mean ± SD)		6.3 ± 6.8	5.5 ± 4.6	P = 0.871***
Sex N (%)	Male	12(35.3)	11(31.4)	P = 0.733*
	Female	22(67.7)	22(68.6)	
Cancer type N (%)	Gastrointestinal	16(47.1)	11(31.4)	P = 0.505**
	Breast	11(32.4)	10(28.6)	
	Lung	3(8.8)	5(14.3)	
	Genital	2(5.9)	5(14.3)	
	Lymphatic	0(0.0)	2(5.7)	
	Bone	2(5.9)	2(5.7)	
Previous chemotherapy experience N (%)	Yes	31(91.2)	34(97.1)	P = 0.298***
	No	3(8.8)	1(2.9)	
Experience laughing yoga N (%)	Yes	1(2.9)	0(0.0)	P = 0.493****
	No	33(97.1)	35(100.0)	

* Chi-square test **Exact Chi-square test ***Mann-Whitney U test ***Fisher’s exact test

At the pre-intervention stage, the mean scores of the physical function, role function, emotional function, cognitive function and social function in the intervention and control groups were not statistically significant (p > 0.05). But at at the post test the mean scores of the three functional domains such as physical functioning, role functioning and emotional functioning in the intervention group was significantly higher than that in the control group respectivly. It means that the mean scores of the physical functioning domain at the post-test in the intervention group (66.27 ± 17.59) was significantly higher than that in the control group (60.57 ± 18.81) (p < 0.05) Also mean scores of the role functioning domain in the intervention group (71.08 ± 25.06) was significantly higher than that in the control group (65 ± 76 ± 25.81) (p < 0.05). And emotional functioning domain in the intervention group (80.88 ± 16.60) was significantly higher than that in the control group (66.19 ± 26.19) (p < 0.05), (Table 2).

**Table 2:** Scores of functional domains of hetalth-related quality of life

Variables		Groups				Intergroup comparison results
		Intervention		Control		
		Mean ± SD	No.	Mean ± SD	No.	
Physical Function	Pre-intervention	65.49 ± 16.32	34	63.81 ± 17.36	35	P = 0.682*
	Post-intervention	66.27 ± 17.59	34	60.57 ± 18.81	35	P = 0.198*
	Pre- and post-intervention difference	0.78 ± 6.08	34	-3.23 ± 4.94	35	P = 0.002**
Intragroup comparison		P = 0.485****		P < 0.001****		
Role Function	Pre-intervention	67.65 ± 26.57	34	65.71 ± 25.86	35	P = 0.805**
	Post-intervention	71.08 ± 25.06	34	65 ± 76 ± 25.81	35	P = 0.337**
	Pre- and post-intervention difference	3.43 ± 7.97	34	-0.95 ± 3.92	35	P = 0.004**
Intragroup comparison		P = 0.020***		P = 0.157***		
Emotional Function	Pre-intervention	67.89 ± 22.01	34	66.19 ± 26.73	35	P = 0.961**
	Post-intervention	80.88 ± 16.60	34	66.19 ± 26.19	35	P = 0.014**
	Pre- and post-intervention difference	12.99 ± 10.49	34	0.00 ± 6.06	35	P.0.001**
Intragroup comparison		P < 0.001****		P = 0.861***		
Cognitive Function	Pre-intervention	81.37 ± 17.77	34	80.48 ± 20.02	35	P = 0.970**
	Post-intervention	79.41 ± 20.94	34	77.62 ± 20.98	35	P = 0.717**
	Pre- and post-intervention difference	-1.96 ± 8.95	34	-2.85 ± 10.29	35	P = 0.783**
Intragroup comparison		P = 0.206***		P = 0.107***		
Social Function	Pre-intervention	76.96 ± 25.62	34	58.57 ± 57.64	35	P = 0.040**
	Post-intervention	77.45 ± 22.05	34	65.71 ± 29.41	35	P = 0.085**
	Pre- and post-intervention difference	0.49 ± 10.44	34	7.14 ± 46.31	35	P = 0.391**
Intragroup comparison		P = 1.000***		P = 0.518***		

*Independent-samples t-test **Mann-Whitney U test ***Wilcoxon test ****Paired-samples t-test

Regarding the symptoms and single items at baseline, the mean scores of the nausea/vomiting, fatigue, pain, dyspnea, sleep, appetite, constipation, diarrhea, financial and HRQOL (overall health and quality of life) in the intervention and control groups were not statistically significant (p > 0.05) At post-test, the mean scores of fatigue and pain in the intervention group were significantly lower than the control group (p < 0.05) (Table 3).The mean scores of the overall QOL status at baseline for the intervention and control groups were not statistically significant (p = 0.167) as well as in the post-test (p = 0.757). However, in the intra-group comparisons, the overall QOL status mean score in the post-intervention stage increased significantly (p < 0.001) (Table 3). No adverse effects were reported by the participants during the study period.

**Table 3:** Scores of symptoms and single items domains of health-related quality of life

Variables		**Groups**				**Intergroup comparison results**
		Intervention		Control		
		Mean ± SD	No.	Mean ± SD	No.	
Nausea/vomiting	Pre-intervention	11.27 ± 19.98	34	22.86 ± 26.22	35	P = 0.032**
	Post-intervention	10.78 ± 20.05	34	23.33 ± 25.94	35	P = 0.013**
	Pre- and post-intervention difference	0.49 ± 5.00	34	0.47 ± 2.81	35	P = 0.321**
Intragroup comparison		P = 0.564***		P = 0.317******		
Fatigue	Pre-intervention	40.52 ± 27.47	34	46.03 ± 28.28	35	P = 0.432**
	Post-intervention	31.70 ± 25.23	34	45.08 ± 27.73	35	P = 0.039**
	Pre- and post-intervention difference	-8.82 ± 22.01	34	-0.95 ± 7.31	35	P < 0.001**
Intragroup comparison		P = 0.001***		P = 0.335******		
Pain	Pre-intervention	27.45 ± 31.48	34	37.62 ± 33.66	35	P = 0.161**
	Post-intervention	19.12 ± 26.31	34	38.57 ± 31.25	35	P = 0.004**
	Pre- and post-intervention difference	-8.33 ± 11.78	34	0.95 ± 11.39	35	P = 0.001**
Intragroup comparison		P = 0.001***		P = 0.603***		
Dyspnea	Pre-intervention	18.63 ± 28.65	34	15.24 ± 27.22	35	P = 0.489**
	Post-intervention	17.65 ± 27.50	34	20.00 ± 31.51	35	P = 1.000**
	Pre- and post-intervention difference	-0.98 ± 5.71	34	4.76 ± 20.03	35	P = 0.102**
Intragroup comparison		P = 0.317***		P = 0.157***		
Sleep disturbance	Pre-intervention	36.27 ± 30.00	34	29.52 ± 36.84	35	P = 0.214**
	Post-intervention	20.59 ± 25.96	34	29.52 ± 35.94	35	P = 0.397**
	Pre- and post-intervention difference	-15.68 ± 18.77	34	0.00 ± 8.08	35	P < 0.001**
Intragroup comparison		P < 0.001***		P = 1.000***		
Loss of appetite	Pre-intervention	32.35 ± 30.13	34	34.29 ± 29.68	35	P = 0.732**
	Post-intervention	30.39 ± 30.00	34	35.24 ± 30.18	35	P = 0.455**
	Pre- and post-intervention difference	-1.96 ± 7.96	34	0.95 ± 5.63	35	P = 0.083**
Intragroup comparison		P = 0.157***		P = 0.317***		
Constipation	Pre-intervention	30.39 ± 37.93	34	37.14 ± 39.41	35	P = 0.482**
	Post-intervention	29.41 ± 36.48	34	38.10 ± 38.89	35	P = 0.372**
	Pre- and post-intervention difference	-0.98 ± 10.00	34	0.95 ± 5.63	35	P = 0.321**
Intragroup comparison		P = 0.564***		P = 0.317***		
Diarrhea	Pre-intervention	15.69 ± 28.70	34	16.19 ± 30.64	35	P = 0.909**
	Post-intervention	13.73 ± 26.10	34	15.24 ± 29.53	35	P = 0.981**
	Pre- and post-intervention difference	-1.96 ± 7.96	34	-0.95 ± 9.58	35	P = 0.650**
Intragroup comparison		P = 0.157***		P = 0.564***		
Financial difficulty	Pre-intervention	54.90 ± 31.65	34	59.05 ± 34.38	35	P = 0.514**
	Post-intervention	51.96 ± 31.98	34	62.86 ± 31.07	35	P = 0.150**
	Pre- and post-intervention difference	-2.94 ± 12.62	34	3.80 ± 22.53	35	P = 0.167**
Intragroup comparison		P = 0.180***		P = 0.366***		
Overall QOL	Pre-intervention	58.58 ± 20.66	34	65.24 ± 19.22	35	P = 0.167**
	Post-intervention	64.95 ± 20.59	34	62.62 ± 18.45	35	P = 0.757**
	Pre- and post-intervention difference	6.37 ± 5.04	34	-2.61 ± 5.25	35	P < 0.001**
Intragroup comparison		P < 0.001***		P = 0.006****		

*Independent-samples t-test **Mann-Whitney U test ***Wilcoxon test ****Paired-samples t-test

HRQOL means participants perspective on their overall health and quality of life

## Discussion

Our study shed light the effect of laughter yoga on HRQOL in cancer patients undergoing chemotherapy. The findings revealed that the implementation of the laughter yoga has improved the cancer patients HRQOL in terms of emotional functioning, role functioning, physical functioning, and overall HRQOL status. Laughter yoga had also reduced the symptoms of fatigue, pain, sleep disturbance, as well as nausea/vomiting. Implementing laughter yoga to the cancer patients undergoing chemotherapy in clinical settings by trained personnel thus might be helpful to alleviate their difficulties and enhance HRQOL.

In a study in Japan, the findings had demonstrated that laughter accompanied by exercises had boosted emotional functioning in older adults. In the present study, laughter yoga had further augmented emotional functioning among the cancer patients thanks to their positive feelings and emotions, which could in turn have a positive effect on emotional functioning [33]. Laughter yoga in our study has improved the role functioning of the cancer patients, since it led to improvements in mental and psychological issues [17] because mental health was often interrelated with physical health status[34], which could significantly contribute to better overall functioning of cancer patients. In another study laughter yoga sessions held twice a week for a month had correspondingly relieved fatigue in cancer patients undergoing external radiotherapy [35]. Laughter can moderate stress hormones, such as cortisol, increase the body’s readiness to cope with various types of stress [36, 37], and eventually eliminate some symptoms, such as fatigue, and thus promote HRQOL.

Likewise, a four-session fun-laughter program in another study had been able to lower pain in patients with rheumatoid arthritis [38]. Laughter yoga in the present study had similarly alleviated pain among cancer patients undergoing chemotherapy. Researches have also demonstrated that laughter therapy could increase pain tolerance and decrease pain perception through physiological mechanisms involving the release of endorphins [39, 40]. According to another study, four laughter yoga sessions had boosted mental well-being in patients undergoing chemotherapy by 6% [23]. Such studies have supported that the four-session laughter yoga program in the present study had enough dose to achieve the outcomes. As evidenced in another study, laughter yoga had elevated sleep quality scores in patients with Parkinson’s disease [25]. Sleep quality in the present study was also enhanced, as one of the most important benefits of laughter was the release of endorphins, a natural painkiller that could reduce pain and fatigue, and enhance sleep quality in patients undergoing chemotherapy [41]. According to a study in Korea, compulsive laughter in the elderly with depression had boosted their sleep quality by 15.5%, and the mean scores of depression had decreased in the laughter group [42]. Laughter was further effective in promoting a positive mood associated with higher HRQOL [36, 38].

In a study done in Turkey, six sessions of breathing exercises had reduced the incidence rate and severity of nausea/vomiting in breast cancer patients receiving chemotherapy [37]. The present study also showed a reduction in nausea/vomiting, possibly due to the role of breathing component of laughter yoga intervention program. Of note, laughter yoga involves deep breathing exercises with hand and foot movements integrated with laughter [43]. Breathing exercises had been found to reduce tension, anxiety, and stress that could be effective in alleviating nausea/vomiting [44]. Moreover, the Manual of Guidelines for Cancer Care to Patients Undergoing Chemotherapy has recommended deep breathing exercises to prevent nausea/vomiting [37, 45]. Shahidi et al. (2011) found that the effect of laughter yoga had decreased the depression mean scores by 60%, compared with the 37.8% reduction in the exercise group in older women [16]. Additionally, another study had reported that patients with higher HRQOL had shown more interest in assuming a role in society, and they mostly had positive emotions and experiences and less depression, which was in line with the present study [46]. It was thus concluded that laughter yoga can be practiced as a treatment option. In the same way, one study had reported that laughter therapy had improved HRQOL in patients with breast cancer undergoing radiotherapy [35], supporting the usefulness of laughter to cancer patients receiving external radiotherapy. In the present study, laughter yoga had elevated emotional functioning, role functioning, and overall HRQOL status as well as the symptoms of fatigue, pain, and sleep disturbance. The laughter yoga, that generally accompanied by appropriate breathing exercises as well as physical movement, had been used to promote health and wellbeing of apparently healthy people and patients with different ailments [14, 17, 20, 21, 43], as a good intervention to mitigate symptoms and improve the overall health and quality of life of cancer patients undergoing chemotherapy. Since the present study was conducted among appropriately selected patients in a clinical setting, it was easy to bring this intervention into real world and clinical practice in healthcare settings that provide chemotherapy.

The possible mechanism of beneficial effect of laughter yoga seen in the interventional group could be due to the effect of laughter yoga on neurological, endocrine, and immune systems of human body. Laughter has shown to cause release of endorphins and dopamine, decrease of cortisol (stress hormone), and affects production of cytokines in the human body. These effect in turn enhances positive mood and sleep, modulates inflammation, and increases pain tolerance. The real or fake laughter is described to exert similar effect in the body [47, 48]. The positive effect on health of laughter yoga could also be partly due to effect of other components of laughter yoga such as movements, breathing, social connection, and affirmations. This could be the reason of decrease in pain and fatigue, and improvement in sleep, role functioning, emotional functioning, physical functioning, and Global health and quality of life of study participants who received laughter yoga intervention [18–21, 36–40, 49, 50].

The present study had some limitations. Firstly, the positive effects observed could partly be due to different factors such as the social circumstances created for the laughter yoga intervention, the competency of the laughter yoga instructor, or due to the environment of hospital, or other patients related factors like the level of self-care, and the type of diet they used. Secondly, in the study, the intervention was administered in a moderate group of 8 to 14 participants, for about 20–30 min, and final outcome was assessed after four sessions. Likewise, the intervention was delivered to the patients undergoing chemotherapy and meeting all eligible criteria that consisted of the patients who could walk to do the laughter yoga in standing posture, further study may be needed to see the effects of laughter among the patients who are currently in wheel chair or in bed but still can laugh and do some physical movement and deep breathing, and on those patients who are using other treatment modalities. Finally, since the outcome measure is based on patient reported outcome as measured by structured tool, EORTC QLQ-C30, there can be chances of recall bias [51]. We suggested since this kind of study reflects subjective experiences of the patients, it would be better to incorporate other tools or technique to obtain objectively measurable data that can indicate the specific effect of the intervention in upcoming researches. Though the participants in this study did not reveal any signs of harms of laughter yoga practice, some literatures suggest that such practice may cause discomforts or harms to some individuals [52].

## Conclusion

This trial showed that a structured laughter yoga intervention program in a hospital setting delivered by trained instructor for 20–30 min each week for four weeks before chemotherapy effectively improve health-related quality of life of the cancer patients undergoing chemotherapy. This intervention showed enhancement of emotional functioning, improvement overall health and quality of life, and mitigation of fatigue, pain, and sleep disturbance symptoms. Many patients could be benefitted if laughter yoga is incorporated as a complementary therapy in routine clinical care practice.

## Electronic supplementary material

Below is the link to the electronic supplementary material.

Supplementary Material 1

## Data Availability

The datasets generated in the present study are available from the corresponding author upon reasonable request.

## Abbreviations

HRQOL: Health-related quality of life
UGI: Upper gastrointestinal
EORTC: European Organization for Research and Treatment of Cancer
MHME: Ministry of Health and Medical Education
WHO: World Health Organization
GH/QOL: Global health and quality of life
