# Supplementary material for: Effects of laughter yoga on health-related quality of life in cancer patients undergoing chemotherapy: a randomized clinical trial
Source: BMC Complement Med Ther. 2023 Jun 12;23:192. doi: 10.1186/s12906-023-04028-2 (PMC10259013; doi:10.1186/s12906-023-04028-2)
Supplement: Supplementary file 1 — Supplementary Material 1 [file 12906_2023_4028_MOESM1_ESM.docx]

Supplementary material 1

**15 Steps of the laughter yoga program**

| Steps | Activities |
| --- | --- |
| 1 | Clapping with the song *1-2-3* ... *1-2-3* and saying *ho-ho-ho* ... *ha-ha-ha* |
| 2 | Taking a deep breath through the nose and a long one through the mouth (five repetitions) |
| 3 | Flexing and stretching the neck and shoulders (five times each side) |
| 4 | Laughing one’s heart out (namely, opening both arms at the top of the head, bending the head slightly backward, and laughing at the same time) |
| 5 | Laughing and saying greetings (viz. placing the palms in front of the face and laughing with eye contact with other people in the group) |
| 6 | Being grateful and laugh (i.e., making a circle with the thumb and forefinger and assuming a state as if one is grateful to the group members and laughing at the same time) |
| 7 | Having one-meter laughter (namely, placing one hand on the other and extending them to the sides and then pulling them by three movements and saying *aaaaa* ... *aaaaa* ... *aaaaa*), after that, the group members laugh one’s heart out with both hands open on both sides and bend their heads slightly backward (four repetitions) |
| 8 | Having silent laughter (viz. opening the mouth as much as possible and laughing without making any sound while looking into the eyes of others and making different utterances at the same time) |
| 9 | Having whispered laughter with closed mouth (i.e., closing the mouth and laughing by whispering and going from place to place at the same time and shaking hands with other group members) |
| 10 | Having cheerful laughter (namely, making a large circle and making the sounds of *aa* ..., *oh oh* ..., *oh* ... *wow* ... and then running toward the center of the circle and laughing) |
| 11 | Having lion laughter (viz. sticking the tongue out and opening the eyes as much as possible and then clasping the hands like a lion in front of the face and laughing one’s your heart out) |
| 12 | Laughing during phone conversation (namely, imagining a phone next to the ear and laughing at each other by making different gestures and nodding at the group members) |
| 13 | Having hassle laughter (i.e., starting laughing by pointing the index finger) |
| 14 | Having gradual laughter (viz. starting with making a smile on the lips, smiling gradually until it turns into loud laughter, and at the end, stopping the laughter gradually) |
| 15 | Having intimacy laughter (namely, approaching each other and holding hands or hugging and laughing at each other). At the end of the sessions, the participants chanted some positive sentences such as “*I am the happiest person on earth*” and “*I am the healthiest person on earth*” |
